# High Patient Satisfaction with a Digital Care Platform After Thoracolumbar Spine Surgery: A Registry-Based Analysis

**DOI:** 10.3390/healthcare14152319

**Published:** 2026-08-01

**Authors:** Phil Bohlen, Nicholas Dietzman, Woo-Keun Kwon, Jannik Leyendecker, Paula Krause, Jan Bredow, Peer Eysel, Albert Telfeian, Peter Derman, Osama Kashlan, Sanjay Konakondla, John Ogunlade, Saqib Hasan, Meng Huang, Mark A. Mahan, Imad Khan, Raymond J. Gardocki, Mark Lambrechts, Anubhav G. Amin, David Huie, Galal Elsayed, Gregory Basil, Christoph P. Hofstetter

**Affiliations:** 1 Department of Neurological Surgery, University of Washington, Seattle, WA 98104, USAchh9045@uw.edu (C.P.H.); 2 Department of Orthopedics and Trauma Surgery, Faculty of Medicine, University Hospital Cologne, University of Cologne, 50937 Cologne, Germany; 3 Department of Neurosurgery, Korea University Guro Hospital, Korea University College of Medicine, Seoul 08308, Republic of Korea; 4 Department of Orthopedics and Trauma Surgery, Krankenhaus Porz am Rhein, University of Cologne, 51149 Cologne, Germany; 5 Department of Neurosurgery, Warren Alpert Medical School, Brown University, Providence, RI 02903, USA; 6 Texas Back Institute, Plano, TX 75093, USA; 7 Department of Neurosurgery, University of Michigan, Ann Arbor, MI 48109, USA; 8 Department of Neurosurgery, Geisinger Neuroscience Institute, Danville, PA 17822, USA; 9 Department of Neurological Surgery, Washington University School of Medicine, St. Louis, MO 63110, USA; 10 Golden State Orthopedics & Spine, Walnut Creek, CA 94598, USA; 11 Houston Methodist Department of Neurosurgery, Houston, TX 77030, USA; 12 Department of Neurosurgery, University of Utah, Salt Lake City, UT 84132, USA; 13 Department of Orthopaedic Surgery, Vanderbilt Health, Nashville, TN 37232, USA; 14 Department of Orthopaedic Surgery, Washington University in St. Louis, St. Louis, MO 63110, USA; 15 Duke Spine Division, Duke University School of Medicine, Durham, NC 27710, USA; 16 Department of Neurological Surgery, Weill Cornell Medicine, New York, NY 10065, USA; 17 Department of Neurosurgery, University of Miami, Miami, FL 33136, USA

**Keywords:** digital health, endoscopic spine surgery, mobile application, patient-reported outcomes, app experience

## Abstract

**Highlights:**

**What are the main findings?**

- Among feedback responders after thoracolumbar spine surgery, 90.8% rated their global app experience as “Very Good,” including many patients with less favorable early clinical recovery.
- Older age was consistently associated with a “Poor” app-experience rating across the multivariable analyses.

**What are the implications of the main findings?**

- Favorable app-experience ratings across different early recovery trajectories suggest that the perceived value of digital postoperative care is not solely explained by clinical recovery.
- Age-adapted onboarding and structured implementation support may improve the experience of older patients using digital postoperative care platforms.

**Abstract:**

**Background/Objectives**: SPINEHealthie is a smartphone-based application enabling remote patient monitoring, care delivery and standardized collection of patient-reported outcome measures (PROMs). This registry-based analysis evaluates patient satisfaction with the platform across a large, heterogeneous spine surgery cohort. **Methods**: A total of 1729 thoracolumbar spine surgery cases were eligible for analysis. Among these, 881 (50.9%) completed the in-app feedback survey and constituted the primary feedback cohort. The global app experience was categorized as “Very Good” or “Poor”. Clinical outcomes, i.e., the Visual Analogue Scale (VAS) for back and leg pain and the Oswestry Disability Index (ODI) were assessed at baseline and in the postoperative period; Δ denotes the change from baseline to two weeks postoperation. **Results**: Among feedback responders, 800 patients (90.8%) rated their app experience as “Very Good”, while 80.8% of 639 patients with surgical outcome data reported that the surgery met their expectations. However, app experience and surgical outcome expectations were assessed using different items at different postoperative time points. Among patients whose surgery did not meet expectations, 84.6% rated their app experience as “Very Good”. Patients with a “Very Good” app-experience rating demonstrated significantly greater improvement in back pain (ΔVAS Back pain 2.94 vs. 2.13, *p* = 0.027), leg pain (ΔVAS Leg pain 3.16 vs. 2.14, *p* = 0.019) and disability (ΔODI 11.00 vs. 2.41, *p* < 0.001). In the primary model, older age, shorter follow-up duration and smaller ODI improvement were associated with an unfavorable app experience; only the association with age remained consistent across sensitivity analyses. **Conclusions**: Among feedback responders, SPINEHealthie was associated with a high proportion of favorable global app-experience ratings. Favorable app-experience ratings remained high, even among patients with unmet surgical expectations or failure to achieve the exploratory two-week ODI-improvement threshold, suggesting that perceived platform value is not solely explained by surgical outcome. Older patients and those with limited functional recovery were identified as subgroups that may benefit from targeted onboarding and engagement strategies.

## 1. Introduction

Recent decades have been marked by the development and rapid expansion of minimally invasive and full-endoscopic techniques, which led to shorter hospitalization times and faster recovery trajectories, and even enabled outpatient spine surgery [1,2,3]. From a scientific perspective, it is essential, especially in such an innovation-driven field, to evaluate upcoming techniques to ensure patient safety and evaluate the effectiveness of each approach. However, conventional outcome assessments, in-person encounters and standard registry-based assessments are often unable to capture longitudinal trajectories, where clinically meaningful recovery unfolds rapidly and predominantly outside the hospital [4]. In routine clinical practice, pain documentation, functional surrogate parameters and other questionnaire-based outcomes are often incomplete and heterogeneous owing to time constraints, inter-observer variability and different clinical priorities [5,6].

To address this gap, the integration of digital health platforms with standardized data capture of the perioperative spine surgery care pathway is crucial [7]. With advances in mobile technology and increasing smartphone literacy, digital musculoskeletal programs, e.g., Hinge Health or Kaia Health, as well as orthopedic postoperative engagement platforms, such as Force Therapeutics, have shown sustained patient engagement [8,9,10]. However, while digital platforms can improve remote patient monitoring and longitudinal patient-reported outcome measure (PROM) collection, their patient-perceived value after spine surgery remains insufficiently characterized. In particular, it remains unclear whether satisfaction with a digital tool primarily tracks favorable surgical recovery or whether favorable app-experience ratings may also occur among patients with less favorable early clinical outcomes.

Building on these developments, SPINEHealthie was designed and launched to address the specific needs of perioperative minimally invasive and endoscopic spine surgery by enabling not only standardized, longitudinal PROMs beyond routine in-person encounters but also offering a care pathway with complication surveillance, virtual follow-up and an asynchronous clinician chat function [11]. After the successful implementation of the digital platform and its standardized data capture framework, the next critical step is to evaluate the generalizability and patient-centered value across different spine surgery cohorts. This includes assessing the platform uptake, user satisfaction and perceived usefulness across different procedure types, age groups and other subgroups, while also determining whether the collected outcome measures capture the full spectrum of postoperative recovery [12].

This study leverages the SPINEHealthie app to perform one of the largest registry-level analyses of digital health satisfaction in spine surgery to date, with the primary aim of identifying patient-level factors associated with unfavorable app experience, hereafter referred to as dissatisfaction and defined as a “Poor” global app-experience rating.

## 2. Materials and Methods

### 2.1. Study Design and Patient Population

This study presents a retrospective analysis of prospectively collected registry data, where we analyzed the patient interactions with the SPINEHealthie app. The data were extracted from the platform’s cloud database under the institutional practice framework. In accordance with HIPAA (Health Insurance Portability and Accountability Act) privacy standards, all patient identifiers are removed in the database. Each participating research site has obtained approval from its local institutional review board. PROMs were collected in accordance with national regulatory requirements and the principles of the 1975 Declaration of Helsinki. Patients were eligible for app enrollment if they were 18 years of age or older, English-speaking and had access to a smartphone. A total of 1979 patients were identified in the registry. A total of 109 patients enrolled in the app without a confirmed surgical procedure at the date of data extraction were removed. Patients undergoing cervical procedures (n = 141) were excluded, as cervical outcomes are assessed with cervical-specific instruments, such as the Neck Disability Index (NDI), Visual Analogue Scale (VAS) Neck and arm pain, which are not directly comparable with lumbar and thoracic outcome measures and can therefore not be meaningfully pooled. Additionally, PROM completeness was substantially lower in the cervical subgroup, precluding meaningful outcome-based analyses.

The final thoracic and lumbar eligibility cohort comprised 1729 patients. Patients were included in the satisfaction analysis cohort if they had completed the in-app feedback survey, which was administered at the two-week postoperative time point and alternatively at day 7, month 1 or month 3. Patients without a completed feedback response were excluded. The study did not include an external control group of patients receiving usual care without SPINEHealthie or using an alternative platform.

### 2.2. Data Extraction and Variables

The SPINEHealthie app is a standardized digital platform designed to facilitate remote postoperative follow-up and longitudinal data acquisition. Patient-reported inputs are transmitted through the application, enabling continuous symptom monitoring and asynchronous patient–provider communication within the postoperative care workflow.

The platform collects baseline demographics such as age, sex, BMI (body mass index), employment and smoking statuses, and comorbidities. Parameters regarding patient clinical status, such as duration of symptoms, or opioid use are also assessed. Surgical details, including procedure type, number of operated levels, surgery duration, blood loss and other surgery characteristics are reported by the operating surgeon. Perioperative and postoperative complications can be assessed by patients and clinicians. PROMs, such as the VAS for back or extremity pain or a surrogate parameter for functional outcome, i.e., the Oswestry Disability Index, are reported by patients at baseline prior to surgery and in the follow-up with high granularity: postoperative days 1–7; 2 weeks; 1, 3 and 6 months; and after 1 and 2 years [13,14]. Global app experience was assessed via a single binary feedback item within the app. Patients were asked to rate their overall app experience as either “Very Good” or “Poor”. No intermediate response options were available. For analysis, responses of “Very Good” were classified as a favorable global app-experience rating, whereas responses of “Poor” were classified as an unfavorable global app-experience rating. This item was not designed to replace validated multidimensional usability or patient experience instruments. SPINEHealthie had previously been evaluated using the validated mHealth App Usability Questionnaire (MAUQ) in a separate prospective cohort. The MAUQ assesses multiple aspects of mobile health usability, including ease of use and satisfaction, interface design and usefulness. It was not administered in the present registry cohort. The binary in-app feedback item was the standardized app-experience measure available in the current registry and was used as a pragmatic, low-burden measure of global app experience.

Separately, a surgical outcome survey asked patients whether the surgical outcome met their expectations (single binary question) at one month postoperation. Cross-tabulation with global app experience was restricted to feedback responders with available surgical outcome survey data.

### 2.3. Statistical Analysis

All statistical analyses and graphical visualizations were performed using R (version 4.5.2). Continuous variables are reported as mean ± standard deviation (SD) for approximately normally distributed variables and as median with interquartile range (IQR) for skewed variables. Categorical variables are reported as counts and percentages. Between-group comparisons for continuous variables were performed using Welch’s *t*-test or the Wilcoxon rank-sum test, as appropriate. Categorical comparisons were performed using Pearson’s χ^2^ test when assumptions were adequate, Fisher’s exact test for sparse 2 × 2 comparisons, or Pearson’s χ^2^ test with simulated *p*-values for sparse R × C comparisons.

The delta between pre- and postoperative scores for the VAS Back and Leg pains, as well as for the ODI, were computed as the difference between the two time points. A positive value indicates an improvement. A two-sided *p*-value < 0.05 was considered statistically significant.

Multivariable logistic regression was used to identify associations with a “Poor” app-experience rating. Covariates were selected based on clinical relevance and were limited to reduce the risk of overfitting, given the small number of “Poor” app-experience ratings. Covariates included age, sex, number of operated levels, ΔVAS Back pain, ΔVAS Leg pain, ΔODI and follow-up duration. Follow-up duration was defined as the number of days between the surgical procedure and the last available PROM entry recorded in the SPINEHealthie platform. Provider-level cluster-robust standard errors were used to account for clustering among 19 operating providers. The primary analysis was based on complete cases. Missingness for all model variables and a comparison of included versus excluded feedback responders are provided in Supplementary Tables S1 and S2. Sensitivity analyses were performed after excluding follow-up duration and after additionally adjusting for procedure category. The results are presented in Supplementary Table S3. Results are reported as adjusted odds ratio (aOR) with 95% confidence interval (CI). Model discrimination was assessed using McFadden’s pseudo-R^2^ and the area under the receiver operating characteristic curve (AUC). Global app experience was cross-tabulated against an ODI MCID achievement and surgical outcome expectations using Pearson’s χ^2^ test. An ODI MCID achievement was defined using a 12.8-point improvement threshold based on the previously published spine surgery literature. Because ODI MCID thresholds are commonly interpreted at later postoperative time points, the two-week MCID analysis was considered an exploratory early functional recovery benchmark rather than a definitive measurement of durable clinical success.

## 3. Results

### 3.1. Cohort Demographics

Among the eligibility cohort, 881 (50.9%) completed the in-app feedback survey and constituted the primary feedback cohort. Of this cohort, the mean age was 56.2 ± 16.1 years, with a larger proportion of male patients (n = 478, 55.4%). The mean body mass index was 29.7 ± 6.8 kg/m^2^. The baseline demographic characteristics stratified by global app-experience rating are shown in Table 1.

Regarding preoperative clinical characteristics, preoperative symptom duration was available for 500 patients (56.8%) with a median duration of 9 months (IQR 4–24 months). Regarding smoking habits, 7.0% were active smokers (n = 43), 65.0% had never smoked and 28.0% were former smokers. Smoking status was missing in 30.3% of all patients. A total of 466 patients reported any existing comorbidity. The most frequent comorbidity was hypertension (n = 266), followed by arthritis (n = 150) and diabetes (n = 102).

Surgical characteristics, including procedure type, operative duration, estimated blood loss, and number of operated levels, are summarized in Table 2. The most common procedures performed were transforaminal endoscopic lumbar discectomy (TELD; n = 266, 30.2%), interlaminar endoscopic lumbar discectomy (IELD; n = 235, 26.7%) and lumbar endoscopic unilateral laminotomy for bilateral decompression (LE-ULBD; n = 191, 21.7%). The full list of all procedure types is shown in Table 2. Of all different surgeries, single-level procedures were predominant (n = 770, 87.4%).

### 3.2. Survey Response and Global App-Experience Ratings

**Figure 1:**
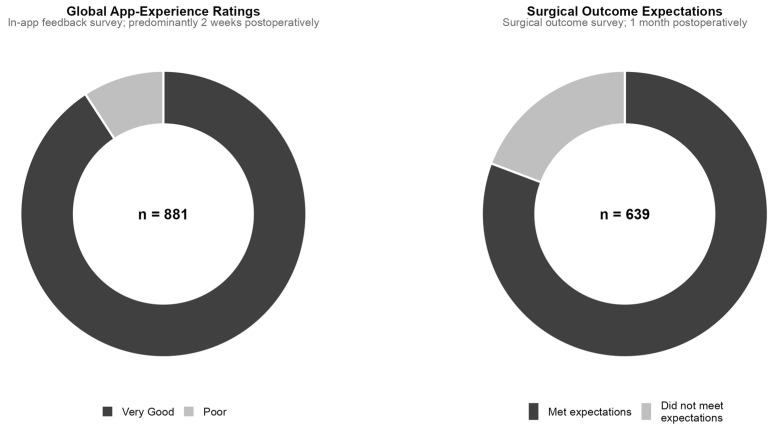
Donut charts depicting global app-experience ratings among feedback responders (left; n = 881, assessed predominantly at two weeks postoperatively) and surgical outcome expectations among feedback responders with available outcome responses (right; n = 639, assessed at one month postoperation).

Among feedback responders, 800 patients (90.8%) rated their overall app experience as “Very Good”, whereas 81 patients (9.2%) rated it as “Poor”. Because 848 of 1729 eligible patients did not complete the feedback survey, these proportions describe the respondent cohort only. The patients most frequently completed their survey at the two-week postoperative time point (n = 802, 91.0%). The second most frequent time point was at one month (n = 60, 6.8%), followed by day 7 (n = 18, 2.0%) and month 3 (n = 1, 0.1%). Separately, the Surgical Outcome Survey was completed by 639 of the feedback patients, of whom 516 (80.8%) reported that the surgery met their expectations, while 123 (19.2%) reported that the surgery did not meet their expectations. The relationship between global app-experience ratings and surgical outcome expectations was further evaluated in stratified analyses (Figure 1).

### 3.3. Comparison by Global App-Experience Rating

Across multiple key dimensions, those patients who rated their app experience as “Very Good” differed significantly from those who rated their app experience as “Poor” (Table 1 and Table 2).

Regarding baseline characteristics, patients with a Very Good app-experience rating were younger than those with a Poor rating (55.4 ± 16.0 vs. 64.4 ± 14.6 years, *p* < 0.001). The sex distribution did not differ significantly between groups (*p* = 0.374).

Preoperative VAS Back pain, VAS Leg pain, and ODI scores were similar between groups, indicating comparable baseline symptom burden and disability. The operative duration did not differ significantly between groups (91.1 ± 51.4 vs. 82.8 ± 52.2 min, *p* = 0.190). The estimated blood loss was low in both groups but differed statistically between groups (5.0 [5.0–10.0] vs. 5.0 [2.0–5.0] mL, *p* = 0.037). However, the distribution of operated levels differed, driven by a higher proportion of ≥3-level procedures in the “Poor” subgroup (6.2% vs. 1.4%) (Table 2).

**Figure 2:**
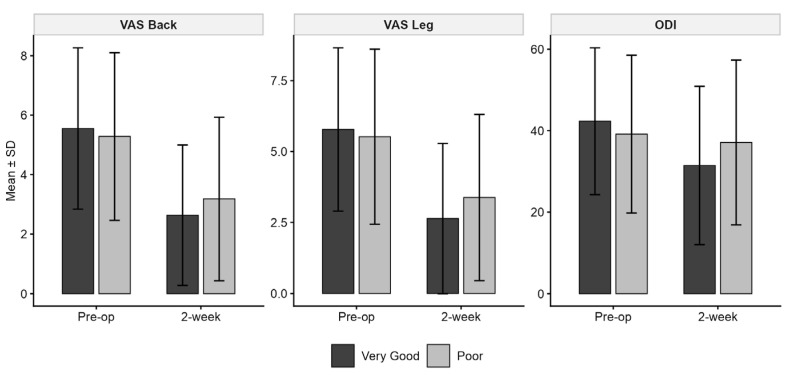
Grouped bar graphs showing mean ± SD of preoperative and two-week postoperative VAS Back pain, VAS Leg pain and ODI scores stratified by app experience rating (“Very Good” vs. “Poor”).

Postoperative clinical outcomes showed greater early improvement among patients with a Very Good app-experience rating (Table 3, Figure 2). Patients with a Very Good rating demonstrated greater improvements in back pain (ΔVAS Back 2.94 ± 3.02 vs. 2.13 ± 2.96, *p* = 0.027), leg pain (ΔVAS Leg 3.16 ± 3.51 vs. 2.14 ± 3.51, *p* = 0.019), and disability (ΔODI 11.00 ± 21.17 vs. 2.41 ± 19.77, *p* < 0.001). Postoperative VAS Leg pain and the postoperative ODI were also lower among patients with a Very Good rating, whereas postoperative VAS Back pain did not differ significantly between groups.

In an exploratory age-stratified analysis, a “Very Good” app-experience rating was reported by 93.9% of patients aged <65 years and 85.7% of patients aged ≥65 years (χ^2^ = 15.19, *p* < 0.001).

### 3.4. Selection and Non-Response Bias Assessment

Among the 1729 eligible thoracolumbar patients, 881 completed the in-app feedback survey and 848 did not. Responders and non-responders were broadly similar with respect to sex, baseline ODI, VAS Back and Leg pains, number of operated levels, operative duration, and procedure region (Table 4). Non-responders were slightly older than responders (57.8 ± 16.2 vs. 56.2 ± 16.1 years, *p* = 0.039). Any comorbidity was more frequently documented among responders than non-responders (83.8% vs. 75.3%, *p* = 0.001). Estimated blood loss differed statistically between groups, although absolute values were low in both groups. The surgical procedure distribution differed significantly between responders and non-responders (*p* < 0.001), indicating potential procedure-related differences in response capture.

### 3.5. Multivariable Adjusted Associations with a “Poor” App-Experience Rating

**Figure 3:**
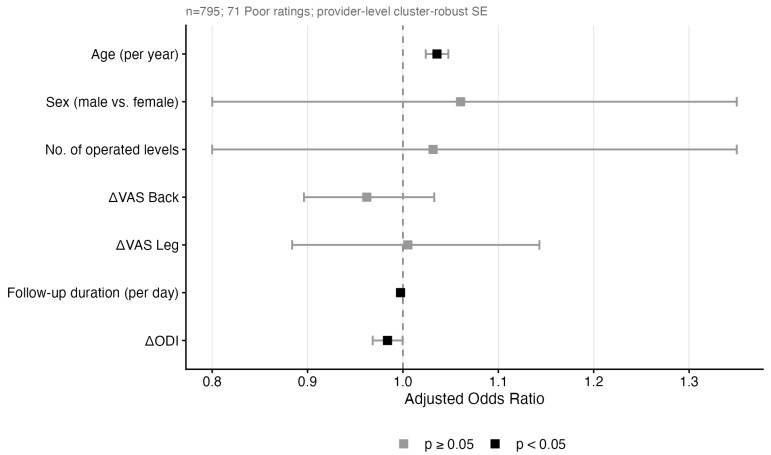
Forest plot of provider-cluster-adjusted associations with a “Poor” app-experience rating. Adjusted odds ratios with 95% confidence intervals are shown for each covariate. Black squares indicate statistically significant associations (*p* < 0.05). Bars indicate confidence intervals extending beyond the displayed axis limit.

Of all feedback cases, 795 patients had a complete dataset for all primary covariates (sex, age, number of operated levels, ΔVAS Back pain, ΔVAS Leg pain, ΔODI and follow-up duration) and were included in the logistic regression model (complete-case analysis, Table 5, Figure 3). The model used provider-level cluster-robust standard errors to account for clustering by operating provider. The model showed moderate discrimination with an AUC of 0.720, but limited explanatory power, with a McFadden’s pseudo-R^2^ of 0.084 (Table 5, Figure 3). In the primary model, three variables were associated with “Poor” app-experience ratings: older age (aOR 1.036 per year, 95% CI 1.024–1.048, *p* < 0.001), shorter follow-up duration (aOR 0.997 per day, 95% CI 0.996–0.999, *p* < 0.001) and lesser functional recovery (aOR 0.984, 95% CI 0.968–1.000, *p* = 0.044).

The other covariates—sex, number of operated levels, ΔVAS Back pain and ΔVAS Leg pain—were not associated with a “Poor” app-experience rating after adjustment.

In sensitivity analyses using provider-level cluster-robust standard errors, older age remained consistently associated with “Poor” app-experience ratings. When follow-up duration was excluded, the association with ΔODI was attenuated and was no longer statistically significant. In the procedure-adjusted sensitivity model, procedure category was not significantly associated with “Poor” app-experience ratings (Supplementary Table S3).

### 3.6. Global App-Experience Ratings Stratified by Clinical Outcome

**Figure 4:**
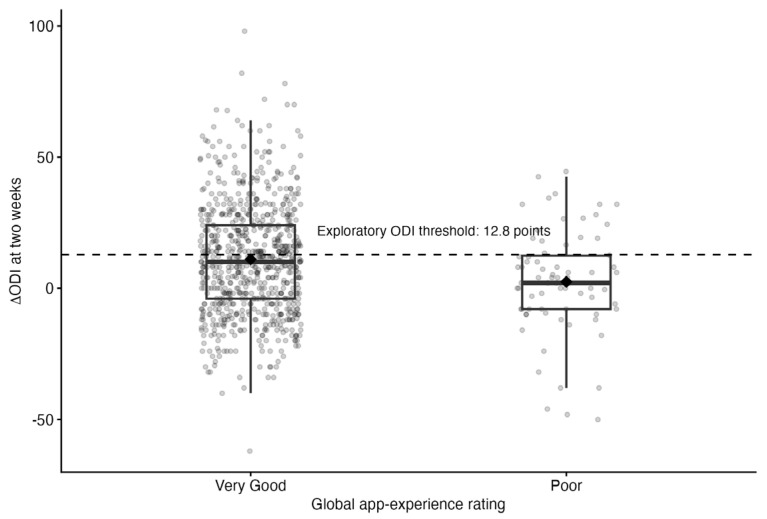
Distribution of early ODI improvement according to global app-experience rating. Each point represents one patient. Points were horizontally jittered to improve visibility. Boxplots show medians and interquartile ranges, diamonds indicate group means, and dashed line represents exploratory 12.8-point ODI improvement threshold. Positive values indicate functional improvement.

The app-experience rating was cross-tabulated against two clinical outcome measures (Table 6) to evaluate whether favorable global app-experience ratings were observed across different early clinical recovery trajectories. Using a 12.8-point ODI improvement threshold as an exploratory early recovery benchmark, 347 patients achieved an ODI MCID at two weeks, whereas 471 did not. Of those patients who failed to achieve the threshold at two weeks, 88.1% rated the app experience as “Very Good”. Among those who achieved an ODI MCID, 94.8% rated the digital health delivery as “Very Good”. The difference between both groups was 6.7% and was statistically significant (χ^2^ = 10.91; *p* < 0.001). Individual ΔODI values showed substantial overlap between rating groups, although the mean early ODI improvement was greater among patients with a “Very Good” rating than among those with a “Poor” app-experience rating (11.00 ± 21.17 vs. 2.41 ± 19.77, *p* < 0.001; Figure 4). These findings should be interpreted as stratification by early functional improvement rather than as a definitive assessment of durable clinical recovery.

The surgical outcome analysis provided an additional descriptive stratification of global app-experience ratings according to whether patients reported that their surgery met their expectations. Among the 123 patients whose surgery did not meet their expectations, 84.6% (n = 104) nevertheless rated their app experience as “Very Good”. Among patients whose surgery met expectations (n = 516), 93.8% rated the app experience as “Very Good” (χ^2^ = 11.56; *p* < 0.001).

## 4. Discussion

This registry-level analysis included 1729 eligible thoracolumbar spine surgery patients, of whom 881 completed the in-app feedback survey. It represents one of the largest published evaluations of digital health platform satisfaction in spine surgery to date. The central finding is that among feedback responders, favorable global app-experience ratings were common, even among patients with less favorable early clinical outcomes. This suggests that the global app-experience rating was not solely aligned with early postoperative recovery, although the single-item endpoint does not permit comprehensive conclusions about digital care satisfaction or usability. Overall, 90.8% of feedback responders rated their experience as “Very Good”. This proportion was numerically higher than the 80.8% of patients with available surgical outcome survey data who reported that their surgery met expectations. However, these outcomes were assessed using different items, at different time points, and in different denominators.

### 4.1. Favorable App-Experience Ratings Despite Suboptimal Clinical Outcomes

This conclusion is reinforced by the stratified analysis. Even among the cohort of patients whose surgery failed to meet their expectations, 84.6% rated their app experience as “Very Good”. This difference reached statistical significance compared with patients whose surgery met expectations (χ^2^ = 11.56, *p* < 0.001). Notably, over 90% of the SPINEHealthie cohort chose to complete the app-feedback survey at the two-week postoperative time point, despite later time points being available. These findings indicate that favorable app-experience ratings were observed, even in patients with unmet surgical expectations or limited early ODI improvement. However, because the app experience endpoint was based on a single forced-choice item, these results should be interpreted as a global patient-reported impressions rather than evidence of comprehensive satisfaction, usability or independent platform value. This finding is consistent with the pilot cohort, where patients described communication with the provider as the most valued app feature [12]. Importantly, that earlier prospective study also evaluated SPINEHealthie using the MAUQ. The app achieved 74.8% of the maximum MAUQ score, including 78.6% for ease of use and satisfaction and 71.4% each for interface design and usefulness. These multidimensional usability findings complement the present registry-level global app-experience ratings and provide additional evidence of favorable patient-perceived experience with SPINEHealthie across different study designs. However, the previous MAUQ analysis was conducted in a smaller, separate cohort after full-endoscopic spine surgery and does not directly validate the binary feedback item used in this study [12,15].

In the past, digital platforms have been evaluated primarily in the context of spine surgery for their feasibility and impact on clinic utilization. Leyendecker et al. demonstrated that the usage of the SPINEHealthie app was associated with higher follow-up compliance and reduced in-person clinic visits after full endoscopic spine surgery [7]. Other data showed lower 90-day readmission and complication rates compared with conventional perioperative care [16]. The comparison between different patient cohorts and age groups also showed a sustained engagement over time with digital platform tools [10]. These data suggest that SPINEHealthie serves not only a functional role for symptom documentation but also for sustained patient–provider contact and reducing care delivery gaps. This may explain why the favorable app-experience ratings remained high, even in the setting of suboptimal clinical outcomes.

### 4.2. Association Between Older Age and “Poor” App-Experience Ratings

The multivariable regression identified older age as the most consistent factor associated with a “Poor” app-experience rating (aOR 1.036 per year, 95% CI 1.024–1.048, *p* < 0.001), which is concordant with the broader digital health literature. A cross-sectional analysis of app experience among chronic disease patients also identified age as a significant predictor of satisfaction alongside employment status and educational level [17]. Additionally, physical and cognitive impairment in the elderly has been shown to predict non-use of digital health tools, also after controlling for sociodemographic variables [18]. These findings together indicate that the relationship between age and satisfaction with digital health tools may reflect barriers related to digital literacy and acceptance, in addition to differences in disease severity or outcome.

Importantly, these findings do not imply that older patients cannot benefit from digital health tools. Rather, these findings identify an implementation challenge: platforms require age-adapted onboarding assistance and simplified interfaces [19]. However, the effect of age on satisfaction should not be interpreted as a fixed biological or generational constant. It is plausible that this described association may attenuate in future cohorts, as individuals who are now middle-aged or younger have been exposed to digital technologies throughout much of their private and professional lives. However, digital health interventions should not solely rely on increasing digital literacy across generations but continue to incorporate accessibility features and structured user support to promote sustained engagement among all patient cohorts.

### 4.3. Functional Recovery and Global App-Experience Ratings

In the primary analysis, greater disability improvement at two weeks was associated with lower odds of a “Poor” app-experience rating (aOR 0.984 per ODI point); however, this association was no longer statistically significant across sensitivity analyses. The mean ΔODI of 11 points in the favorable app-rating subgroup approximated the published MCID for the ODI in lumbar surgery, with 44.2% of patients with a favorable app-experience rating among those with available ODI MCID data achieving this published threshold [20]. In contrast the “Poor” rating group demonstrated a mean ΔODI of only 2.41 points in the ODI, which is effectively no functional improvement at two weeks, compared with 11.00 points in the “Very Good” rating subgroup. While the observational design does not allow causal conclusions, the consistency of this descriptive analyses in not only the continuous variables but also in the MCID-based model supports its clinical relevance: patients with greater early functional improvement were more likely to engage positively with the digital care platform; however, the adjusted ΔODI association was not consistent across sensitivity analyses. Beyond the reported disability outcomes, patients with a favorable app-experience rating also demonstrated significantly lower extremity pain (2.64 ± 2.65 vs. 3.38 ± 2.93, *p* = 0.036), suggesting that leg pain relief may be an additional factor associated with app experience ratings in this cohort.

### 4.4. Practice-Level Engagement and Implementation Implications

The observed surgeon-level heterogeneity in response rates provides a clinically actionable insight beyond the non-response bias assessment itself. The different study sites differed substantially in enrollment practices. Although site-level onboarding workflows were not formally measured, the observed provider-level variation suggests that implementation infrastructure may influence patient engagement. Findings from a companion cohort study demonstrated that early postoperative adherence during the first postoperative week was a factor associated with sustained long-term follow-up [21].

This is further consistent with the broader digital health implementation literature, which identifies structured onboarding and institutional commitment as the primary drivers of mobile health platform uptake, independent of patient demographics or digital literacy [10,22].

The concentration of non-response at the practice-site level suggests that engagement is not only determined by patient-level factors such as older age or lower digital literacy. It further shows that implementation of SPINEHealthie across additional sites will require not only technological deployment but also workflow support, research coordination infrastructure, and surgeon-site engagement monitoring to generate lasting data completeness and outcome interpretability [21,23,24].

### 4.5. Limitations

Several limitations need to be addressed. A key limitation of this study is that the primary app-experience endpoint was based on a single forced-choice global feedback item rather than a validated multidimensional instrument. The forced-choice structure of the app experience may have inflated the proportion of “Very Good” responses, as no intermediate responses were available to choose from. Therefore the endpoint should be interpreted as a crude global impression of app experience and not as a comprehensive assessment of digital care satisfaction. Future studies should incorporate validated multidimensional instruments alongside registry-based clinical outcomes.

Another important limitation is the non-response rate in the in-app feedback survey. Only 881 of 1729 eligible patients completed the primary app-experience endpoint. Patients who were more engaged with the app, may have encountered fewer technical barriers, received stronger provider encouragement or had a more favorable early app-experience rating were more likely to complete the survey. Consequently, the 90.8% favorable app experience may overestimate the true favorable rating rate in the overall cohort and the reported app-experience outcome should not be generalized to all eligible patients. A total of 86 feedback responders with missing covariates were excluded in the multivariable analysis. Although included and excluded cases were compared, bias related to complete-case analysis cannot be excluded.

This registry-based design limits causal inference and residual confounding by unmeasured variables cannot be excluded. This includes baseline expectations, health literacy, prior experience with digital health tools, provider responsiveness, the intensity of onboarding and socioeconomic factors. The 49.1% non-response rate introduces a potential selection bias. The absence of a control group precludes any conclusion about the independent effect of SPINEHealthie compared with usual postoperative care. Expectancy and other effects related to engagement cannot be excluded.

Cervical spine procedures were excluded, as the functional surrogate, i.e., NDI neck and arm pain PROMs, were only available in a few patients. Additionally, a comparison with lumbar-specific instruments is not directly feasible. The present findings should be interpreted as applying primarily to lumbar and thoracic cases. A dedicated analysis of the cervical cohort is warranted in future studies.

The logistic regression accounted for provider-level clustering using cluster-robust standard errors. Nevertheless, residual confounding related to unmeasured provider- and site-level characteristics cannot be excluded. Besides this, the multivariable model had only moderate discrimination and limited explanatory power. Therefore the model should be interpreted as identifying associations with unfavorable app-experience ratings, rather than as providing a strong prediction.

The surgical outcome survey was completed at one month postoperation, whereas the global app-experience ratings and ODI MCID achievement were assessed at two weeks. This short temporal discrepancy between these measures must be acknowledged. Besides that, the use of the ODI MCID at the two-week time point is another limitation, as it is more commonly interpreted at a later postoperative follow-up. Applying it in an earlier recovery phase may not fully capture stable or durable functional recovery.

Follow-up duration was a registry-derived postoperative observation-time variable rather than a baseline characteristic and may reflect engagement, provider workflow or attrition. Therefore its association with app-experience should be interpreted cautiously and not as an independent patient-level predictor.

## 5. Conclusions

Among feedback responders, SPINEHealthie was associated with a high proportion of favorable global app-experience ratings after thoracolumbar spine surgery. Because only 50.9% of eligible patients completed the feedback survey, these findings should be interpreted as respondent-level evidence and may overestimate favorable app-experience in the full eligible cohort. Favorable app-experience ratings were still observed in a substantial proportion of patients with unmet surgical expectations or failure to achieve the early ODI improvement threshold, suggesting that the global app rating was not solely aligned with early clinical recovery. Lower early functional recovery two weeks after surgery, as well as older age and shorter follow-up duration, were associated with unfavorable app-experience ratings and may help identify patient subgroups that could benefit from targeted onboarding, technical support and engagement strategies. However, associations with lower early recovery and shorter follow-up duration were not robust across sensitivity analyses and should be interpreted cautiously. Taken together, these findings support the feasibility and potential patient-perceived value of digital perioperative care delivery platforms in thoracolumbar spine surgery while also identifying clear directions for future development.

## Figures and Tables

**Table 1:** Baseline demographic characteristics of favorable vs. unfavorable app-experience ratings.

	All (n = 881)	“Very Good” (n = 800)	“Poor” (n = 81)	*p*-Value
***N***	***881***	***800***	***81***	
**Age (years)**	56.2 ± 16.1	55.4 ± 16.0	64.4 ± 14.6	<0.001
**Sex**				0.374
Male	478 (55.4%)	430 (54.8%)	48 (60.8%)	
Female	385 (44.6%)	354 (45.2%)	31 (39.2%)	
**BMI (kg/m^2^)**	29.7 ± 6.8	29.8 ± 6.7	29.3 ± 7.5	0.621
**Symptom duration (months), median [IQR]**	9 [4–24]	9 [4–24]	12 [6–30]	0.191
**Comorbidities**				
Any comorbidity	466 (83.8%)	415 (83.7%)	51 (85.0%)	0.937
Hypertension	266 (47.8%)	231 (46.6%)	35 (58.3%)	0.113
Arthritis	150 (27.0%)	133 (26.8%)	17 (28.3%)	0.923
Diabetes mellitus	102 (18.3%)	94 (19.0%)	8 (13.3%)	0.376
Asthma	70 (12.6%)	67 (13.5%)	3 (5.0%)	0.095
Hyperlipidemia	53 (9.5%)	49 (9.9%)	4 (6.7%)	0.570
Coronary artery disease	21 (3.8%)	19 (3.8%)	2 (3.3%)	1.000
Missing	325 (36.9%)	304 (38.0%)	21 (25.9%)	
**Employment status**				0.056
Employed	483 (57.2%)	447 (58.1%)	36 (48.0%)	
Unemployed	35 (4.1%)	34 (4.4%)	1 (1.3%)	
Retired	235 (27.8%)	203 (26.4%)	32 (42.7%)	
Disabled	68 (8.0%)	63 (8.2%)	5 (6.7%)	
Other	24 (2.8%)	23 (3.0%)	1 (1.3%)	
**Smoking status**				0.515
Never	399 (65.0%)	366 (65.6%)	33 (58.9%)	
Active	43 (7.0%)	38 (6.8%)	5 (8.9%)	
Former	172 (28.0%)	154 (27.6%)	18 (32.1%)	

Data presented as mean ± SD, median [IQR], or n (%), as appropriate. Percentages for variables with missing data were calculated using patients with available data; missing counts are reported separately. *p*-values: Continuous variables by Welch’s *t*-test, symptom duration by Wilcoxon rank-sum test, and categorical variables by Pearson’s χ^2^ test or Fisher’s exact test for sparse comparisons. Missing data: Sex (n = 18), comorbidity (n = 325), employment status (n = 36), and smoking status (n = 267). BMI = body mass index; SD = standard deviation; IQR = interquartile range.

**Table 2:** Surgical characteristics of favorable vs. unfavorable app-experience ratings.

	All (n = 881)	“Very Good” (n = 800)	“Poor” (n = 81)	*p*-Value
***N***	***881***	***800***	***81***	
**Operative duration, min**	90.3 ± 51.5	91.1 ± 51.4	82.8 ± 52.2	0.190
**Estimated blood loss, mL**	5.0 [5.0–10.0]	5.0 [5.0–10.0]	5.0 [2.0–5.0]	0.037
**No. of operated levels**				0.010
1	770 (87.4%)	704 (88.0%)	66 (81.5%)	
2	95 (10.8%)	85 (10.6%)	10 (12.3%)	
≥3	16 (1.8%)	11 (1.4%)	5 (6.2%)	
**Surgical procedure**				0.145
TELD	266 (30.2%)	236 (29.5%)	30 (37.0%)	
IELD	235 (26.7%)	223 (27.9%)	12 (14.8%)	
LE-ULBD	191 (21.7%)	172 (21.5%)	19 (23.5%)	
IE LRD	52 (5.9%)	50 (6.2%)	2 (2.5%)	
TELF	51 (5.8%)	45 (5.6%)	6 (7.4%)	
TETD	44 (5.0%)	38 (4.8%)	6 (7.4%)	
Endo-TLIF	16 (1.8%)	14 (1.8%)	2 (2.5%)	
Others	26 (3.0%)	22 (2.8%)	4 (4.9%)	

Data presented as mean ± SD, median [IQR], or n (%), as appropriate. Percentages for variables with missing data were calculated using patients with available data; *p*-values: Operative duration by Welch’s *t*-test; estimated blood loss by Wilcoxon rank-sum test; and categorical variables by Pearson’s χ^2^ test, Fisher’s exact test for sparse 2 × 2 comparisons, or Pearson’s χ^2^ test with simulated *p*-values for sparse R × C. Others include EELD, ICELF, PCFD, lumbar MIS-ULBD, TE-ULBD, and TE LRD. SD = standard deviation; IQR = interquartile range. TELD = transforaminal endoscopic lumbar discectomy; IELD = interlaminar endoscopic lumbar discectomy; LE-ULBD = lumbar endoscopic unilateral laminotomy for bilateral decompression; IE LRD = interlaminar endoscopic lateral recess decompression; TELF = transforaminal endoscopic lumbar foraminotomy; TETD = transforaminal endoscopic thoracic discectomy; Endo-TLIF = endoscopic transforaminal lumbar interbody fusion.

**Table 3:** Clinical outcomes at two weeks by app experience rating.

	All (n = 881)	“Very Good” (n = 800)	“Poor” (n = 81)	*p*-Value
***N (thoracolumbar)***	***881***	***800***	***81***	
**VAS Pain Scores (0–10)**				
Pre-op VAS Back	5.53 ± 2.72	5.55 ± 2.72	5.28 ± 2.82	0.425
Post-op VAS Back (2-week)	2.69 ± 2.40	2.64 ± 2.36	3.18 ± 2.75	0.096
ΔVAS Back (improvement)	2.87 ± 3.02	2.94 ± 3.02	2.13 ± 2.96	0.027
Pre-op VAS Leg	5.76 ± 2.90	5.78 ± 2.88	5.52 ± 3.09	0.485
Post-op VAS Leg (2-week)	2.71 ± 2.68	2.64 ± 2.65	3.38 ± 2.93	0.036
ΔVAS Leg (improvement)	3.07 ± 3.52	3.16 ± 3.51	2.14 ± 3.51	0.019
**Disability (ODI, %)**				
Pre-op ODI (%)	42.04 ± 18.17	42.33 ± 18.03	39.15 ± 19.40	0.171
Post-op ODI (%) (2-week)	31.97 ± 19.57	31.45 ± 19.43	37.10 ± 20.24	0.021
ΔODI (improvement)	10.22 ± 21.18	11.00 ± 21.17	2.41 ± 19.77	<0.001

Data presented as mean ± SD. Postoperative values refer to the two-week postoperative time point. *p*-values by Welch’s *t*-test (“Very Good” vs. “Poor” app-experience rating). ODI = Oswestry Disability Index; VAS = Visual Analogue Scale; SD = standard deviation.

**Table 4:** Comparison of responders and non-responders.

Characteristic	Responders (n = 881)	Non-Responders (n = 848)	Statistical Test	*p*-Value
***N***	***881***	***848***		
**Age, years**	56.2 ± 16.1	57.8 ± 16.2	Welch’s t = −2.06	0.039
**Sex**			χ^2^ = 1.46, dof = 1	0.226
Male	478 (55.4%)	482 (58.4%)		
Female	385 (44.6%)	343 (41.6%)		
**Baseline patient-reported outcomes**				
Pre-op VAS Back	5.5 ± 2.7	5.5 ± 2.8	Welch’s t = −0.05	0.958
Pre-op VAS Leg	5.8 ± 2.9	5.6 ± 3.0	Welch’s t = 0.86	0.392
Pre-op ODI (%)	42.0 ± 18.2	40.4 ± 19.5	Welch’s t = 1.49	0.137
**Any comorbidity**	466 (83.8%)	332 (75.3%)	χ^2^ = 10.67, dof = 1	0.001
**Surgical characteristics**				
**No. of operated levels**	1.15 ± 0.47	1.13 ± 0.38	Welch’s t = 0.91	0.361
**Procedure extent**			χ^2^ = 0.08, dof = 1	0.773
Single level	770 (87.4%)	746 (88.0%)		
Multi-level (≥2)	111 (12.6%)	102 (12.0%)		
**Operative duration, min**	97.6 ± 47.5	96.9 ± 54.4	Welch’s t = 0.15	0.879
**Estimated blood loss, mL**	5.0 [5.0–10.0]	5.0 [5.0–10.0]	Wilcoxon rank-sum	0.044
**Procedure region**			χ^2^ = 0.76, dof = 1	0.384
Lumbar	837 (95.0%)	814 (96.0%)		
Thoracic	44 (5.0%)	34 (4.0%)		
**Surgical procedure**			χ^2^ = 40.36, dof = 7	<0.001
TELD	266 (30.2%)	315 (37.1%)		
IELD	235 (26.7%)	209 (24.6%)		
LE-ULBD	191 (21.7%)	142 (16.7%)		
IE LRD	52 (5.9%)	25 (2.9%)		
TELF	51 (5.8%)	44 (5.2%)		
TETD	44 (5.0%)	34 (4.0%)		
Endo-TLIF	16 (1.8%)	14 (1.7%)		
Others	26 (3.0%)	65 (7.7%)		

Data are presented as mean ± SD, median [IQR], or n (%), as appropriate. Percentages for variables with missing data were calculated using patients with available data. Continuous variables were compared using Welch’s *t*-test; skewed continuous variables using the Wilcoxon rank-sum test; and categorical variables using Pearson’s χ^2^ test, Fisher’s exact test for sparse 2 × 2 comparisons, or Pearson’s χ^2^ test with simulated *p*-values for sparse R × C comparisons. Baseline PROMs and comorbidity status were obtained from the linked clinical dataset. VAS = Visual Analogue Scale; ODI = Oswestry Disability Index; SD = standard deviation; IQR = interquartile range. TELD = transforaminal endoscopic lumbar discectomy; IELD = interlaminar endoscopic lumbar discectomy; LE-ULBD = lumbar endoscopic unilateral laminotomy for bilateral decompression; IE LRD = interlaminar endoscopic lateral recess decompression; TELF = transforaminal endoscopic lumbar foraminotomy; TETD = transforaminal endoscopic thoracic discectomy; Endo-TLIF = endoscopic transforaminal lumbar interbody fusion.

**Table 5:** Cluster-adjusted multivariable logistic regression for “Poor” app-experience rating.

Variable	aOR	95% CI	*p*-Value
**Cluster-adjusted primary model (n = 795; 71 Poor ratings; 19 provider clusters)**			
Age (per year)	1.036	1.024–1.048	**<0.001**
Sex (male vs. female)	1.060	0.651–1.727	0.814
No. of operated levels	1.032	0.620–1.718	0.905
ΔVAS Back	0.962	0.896–1.033	0.286
ΔVAS Leg	1.005	0.884–1.143	0.937
Follow-up duration (per day)	0.997	0.996–0.999	**<0.001**
ΔODI	0.984	0.968–1.000	**0.044**

McFadden R^2^ = 0.084, AUC = 0.720. aOR = adjusted odds ratio for a “Poor” app-experience rating. Standard errors, 95% confidence intervals, and *p*-values were calculated using provider-level cluster-robust variance estimation. Complete-case analysis; 86 patients excluded because of missing covariate or provider-cluster data. VAS = Visual Analogue Scale; ODI = Oswestry Disability Index.

**Table 6:** Global app-experience ratings stratified by clinical outcome.

Subgroup	N	“Very Good” App Rating	“Poor” App Rating	*p*-Value
**ODI MCID (2-week)**				χ^2^ = 10.91; *p* < 0.001
MCID not achieved (<12.8)	471	415 (88.1%)	56 (11.9%)	
MCID achieved (≥12.8)	347	329 (94.8%)	18 (5.2%)	
**Surgical Outcome Survey**				χ^2^ = 11.56; *p*< 0.001
Surgery met expectations	516	484 (93.8%)	32 (6.2%)	
Surgery did not meet expectations	123	104 (84.6%)	19 (15.4%)	

App-experience rating was assessed as “Very Good” vs. “Poor”. ODI MCID was evaluated at two weeks; surgical outcome survey was performed at 1 month. ODI MCID threshold: 12.8 points; *p*-values by Pearson’s χ^2^-test. MCID = minimum clinically important difference; ODI = Oswestry Disability Index.

## Data Availability

Restrictions apply to the availability of these data. Data were obtained from Endoscopic Spine Research Group (ESRG) and are available from the authors at www.esrg.net with the permission of the ESRG.

## Supplementary Materials

The following supporting information can be downloaded at: https://www.mdpi.com/article/10.3390/healthcare14152319/s1, Table S1. Missingness of model variables. Table S2. Complete-case included vs. excluded feedback responders. Table S3. Cluster-adjusted sensitivity models for poor app-experience rating.
