# Supplementary material for: High Patient Satisfaction with a Digital Care Platform After Thoracolumbar Spine Surgery: A Registry-Based Analysis
Source: Healthcare (Basel). 2026 Aug 1;14(15):2319. doi: 10.3390/healthcare14152319 (PMC13465706; doi:10.3390/healthcare14152319)
Supplement: Supplementary file 1 [file healthcare-14-02319-s001.zip › healthcare-4381281-supplementary.pdf]

Supplementary Table S1. Missingness of Model Variables

Variable	Available	Missing	Missing [%]
Poor app-experience rating	881	0	0.0%
Age	862	19	2.2%
Sex	863	18	2.0%
No. of operated levels	881	0	0.0%
ΔVAS Back	834	47	5.3%
ΔVAS Leg	834	47	5.3%
Follow-up duration	881	0	0.0%
ΔODI	818	63	7.2%

Missingness is shown among feedback responders included in the candidate regression cohort (n=881).

Supplementary Table S2. Complete-Case Included vs. Excluded Feedback Responders

Variable	Included complete-case (n=795)	Excluded due to missing covariates (n=86)	p-value
N	***795***	***86***	
**App-experience rating**			0.531
Very Good	724 (91.1%)	76 (88.4%)	
Poor	71 (8.9%)	10 (11.6%)	
**Age, years**	56.2 ± 16.0	56.8 ± 17.8	0.770
**Sex**			0.767
Male	442 (55.6%)	36 (52.9%)	
Female	353 (44.4%)	32 (47.1%)	
**No. of operated levels**			0.020
1	698 (87.8%)	72 (83.7%)	
2	86 (10.8%)	9 (10.5%)	
≥3	11 (1.4%)	5 (5.8%)	
**VAS Pain Scores**			
Pre-op VAS Back	5.52 ± 2.73	5.59 ± 2.68	0.863
Post-op VAS Back (2-week)	2.66 ± 2.37	3.08 ± 2.78	0.262

Variable	Included complete-case (n=795)	Excluded due to missing covariates (n=86)	p-value
ΔVAS Back	2.87 ± 3.04	2.92 ± 2.62	0.910
Pre-op VAS Leg	5.79 ± 2.89	5.34 ± 2.95	0.267
Post-op VAS Leg (2-week)	2.66 ± 2.64	3.33 ± 3.15	0.120
ΔVAS Leg	3.12 ± 3.48	2.02 ± 4.22	0.114
**ODI**			
Pre-op ODI (%)	42.15 ± 18.12	40.15 ± 19.08	0.492
Post-op ODI (%) (2-week)	31.81 ± 19.24	34.22 ± 23.66	0.451
ΔODI	10.34 ± 21.26	6.24 ± 18.05	0.297
**Follow-up duration, days**	221.5 ± 212.2	130.3 ± 185.1	<0.001

Included patients were complete cases for all variables in the multivariable logistic regression model. Excluded patients were feedback responders with at least one missing model covariate. Continuous variables are presented as mean ± SD and compared using Welch's t-test. Categorical variables are presented as n (%) among available data and compared using Pearson's χ^2-test, Fisher's exact test for sparse 2×2 comparisons, or Pearson's χ^2-test with simulated p-values for sparse RxC comparisons.

Supplementary Table S3. Cluster-Adjusted Sensitivity Models for Poor App-Experience Rating

Variable	aOR	95% CI	p-value
**Sensitivity model excluding follow-up duration (n=795; 71 Poor ratings; 19 provider clusters)**			
Age (per year)	1.033	1.020–1.046	**<0.001**
Sex (male vs. female)	1.126	0.691–1.835	0.635
No. of operated levels	1.040	0.629–1.720	0.879
ΔVAS Back	0.948	0.883–1.017	0.136
ΔVAS Leg	1.007	0.889–1.140	0.918
ΔODI	0.986	0.971–1.002	0.079
**Procedure-adjusted sensitivity model (n=795; 71 Poor ratings; 19 provider clusters)**			
Age (per year)	1.037	1.026–1.048	**<0.001**
Sex (male vs. female)	1.134	0.692–1.860	0.618
No. of operated levels	1.102	0.686–1.770	0.688
ΔVAS Back	0.953	0.884–1.028	0.212
ΔVAS Leg	1.002	0.879–1.142	0.980
ΔODI	0.985	0.969–1.001	0.064
Procedure: decompression/foraminotomy vs. discectomy	0.674	0.353–1.288	0.232

Variable	aOR	95% CI	p-value
Procedure: fusion/other vs. discectomy	0.694	0.333–1.446	0.330

All models used provider-level cluster-robust standard errors. aOR = adjusted odds ratio for a “Poor” app-experience rating. The procedure-adjusted model used discectomy as the reference category.
